# Plastic Classification Using Optical Parameter Features Measured with the TMF8801 Direct Time-of-Flight Depth Sensor

**DOI:** 10.3390/s23063324

**Published:** 2023-03-22

**Authors:** Cienna N. Becker, Lucas J. Koerner

**Affiliations:** Department of Computer and Electrical Engineering, University of St. Thomas School of Engineering, St. Paul, MN 55105, USA

**Keywords:** material sensing, material classification, material impulse response function (MIRF), time of flight (ToF)

## Abstract

We demonstrate a methodology for non-contact classification of five different plastic types using an inexpensive direct time-of-flight (ToF) sensor, the AMS TMF8801, designed for consumer electronics. The direct ToF sensor measures the time for a brief pulse of light to return from the material with the intensity change and spatial and temporal spread of the returned light conveying information on the optical properties of the material. We use measured ToF histogram data of all five plastics, captured at a range of sensor to material distances, to train a classifier that achieves 96% accuracy on a test dataset. To extend the generality and provide insight into the classification process, we fit the ToF histogram data to a physics-based model that differentiates between surface scattering and subsurface scattering. Three optical parameters of the ratio of direct to subsurface intensity, the object distance, and the time constant of the subsurface exponential decay are used as features for a classifier that achieves 88% accuracy. Additional measurements at a fixed distance of 22.5 cm showed perfect classification and revealed that Poisson noise is not the most significant source of variation when measurements are taken over a range of object distances. In total, this work proposes optical parameters for material classification that are robust over object distance and measurable by miniature direct time-of-flight sensors designed for installation in smartphones.

## 1. Introduction

Material classification, or the means of identifying the substance of an object, has broad applications. In education, material classification could assist young children in identifying objects. In health, this technology may give those who are blind or poor-sighted a new way to “see” their surroundings. In media and entertainment, it could bring the television experience to new heights, and turn video gaming into a 3D, immersive experience, just like one study predicts [1]. In industrial settings, material classification may guide sorting for assembly or recycling.

There are many unique approaches to nondestructive material classification. Physical characteristics, such as elasticity [2], water permeability, and thermal imagery [3] have been used, but tend to require advanced hardware, customized system set-ups, or specific environments. The benefits of optical properties as a means of identification are numerous, including the possibility of remote classification nearing conventional video rates of 30 frames per second. Hyperspectral imaging captures the distinct spectral signatures of materials to determine object composition with excellent sensitivity. Hyperspectral imaging is used for material classification in applications such as food safety, industrial sorting, medical diagnostics, and precision agriculture [4]. These instruments require a specialized camera and broadband illumination, with most commercial systems being expensive and bulky [5]. However, progress is being made toward miniaturized sensors [6] and hand-held commercial hyperspectral instruments. As an example, the commercially available hand-held Specim IQ camera is still relatively bulky with a weight of 1.3 kg and a required external illumination source [7]. Material classification by analysis of RGB images [8,9] fails when presented with substances of similar visual surface appearance or 2D printed copies, partly because texture is not directly measured. An alternative optical approach has emerged that classifies materials based on parameters measured by time-of-flight (ToF) depth sensors. The transient optical response of a material, most commonly termed the material impulse response function (MIRF) or the temporal point spread function (TPSF), relates to the surface and subsurface scattering properties of the material, and so provides a rich set of information to distinguish materials. The material classification capabilities of ToF sensors warrants investigation, partly because smartphones are driving developments that miniaturize and reduce the cost of these devices.

ToF sensors are distinguished by the approach used to modulate the illumination source. Indirect ToF sensing illuminates the scene with a continuous wave and detects the phase shift that accumulates as the light travels to and back from the objects to measure object depth. The most well-known indirect ToF sensor may be the Microsoft Kinect. Material classification enabled by indirect ToF was demonstrated to classify visually similar materials of paper, styrofoam, towel, and wax using a Photonic Mixing Device (PMD) camera with an 80.9% accuracy level without dependence on depth [10]. These measurements required a high-powered illumination source of six 250 mW laser diodes at 650 nm wavelength [11]. The work of Tanaka et al. captures depth distortions using a Microsoft Kinect v2 ToF camera to characterize a set of 24 different materials with 85.8% accuracy, yet requiring measurements over a range of distances (600 mm to 1200 mm) and over three modulation frequencies [12]. If the distance dependence of the depth distortions is not available as a feature, the classification accuracy degrades to 55.0% [12]. Recently, improvements to the efficiency of sensing the MIRF using indirect ToF sensors were developed that used direct Fourier sampling [13]. The classifier achieves 78% accuracy at the pixel level and 98% when neighboring measurements are grouped into superpixels, and shows the possibility of frame rates that exceed 10 per second [13].

Direct ToF sensors illuminate the scene with a short laser pulse with the returning photons detected by single-photon avalanche photodiodes (SPADs) and the arrival time measured by a time-to-digital converter (TDC). Photon return times are accumulated over many laser pulses to construct a timing histogram that is processed to determine object depth or other transient features, such as subsurface scattering. SPADs integrated with CMOS electronics create miniature and low-power depth sensors that benefit from CMOS scaling; recently, time-gated SPAD sensors have been demonstrated at mega-pixel formats [14]. Nearly every smartphone has an SPAD-based proximity sensor to disable touch screen input when the phone is placed to an ear, and some now include a longer-range multi-pixel direct ToF depth sensor [15]. Since these sensors are designed for smartphones, the system is low-power and the illumination is eye-safe. Callenberg et al. used a commercially available direct ToF sensor (ST VL53L1X [16]) to classify materials [17]. The sensor is placed in direct contact, and the return time histogram data are used as features to train a convolutional neural network that identifies foam, paper, skin, towel, and wax with an accuracy greater than 90% [17]. By placing the sensor in direct contact with the material and varying the region of interest (ROI) of the detector, this system is sensitive to angular and spatial variations of the returned optical transient. The timing resolution of the ST VL53L1X is rather coarse, with only 24 histogram bins and a bin size of around 1000 ps.

**Figure 1:**
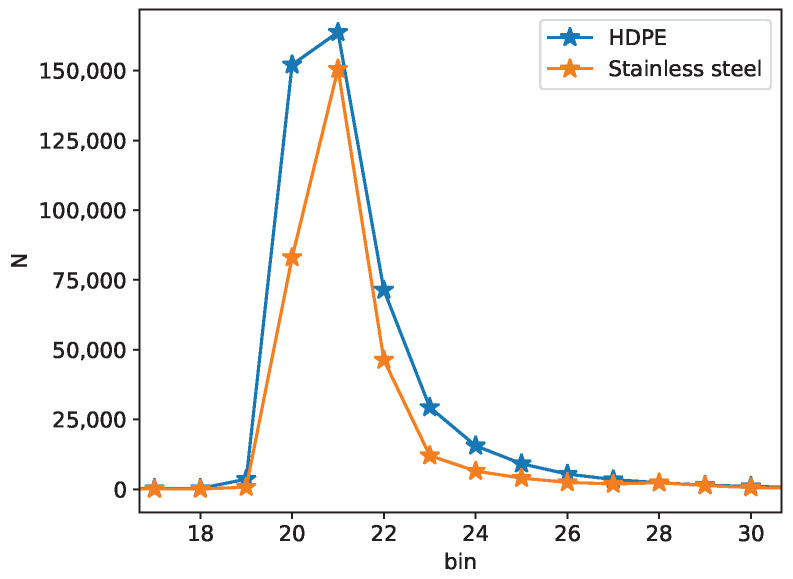
Two measured ToF histograms using a plastic sample (HDPE) and stainless steel. The stainless steel has minimal subsurface scattering, while the HDPE demonstrates a noticeable tail.

In this work, we present material classification using the AMS TMF8801 direct time-of-flight sensor with a price of $3 at large quantities, an active power consumption of only 81 mW, and a longest dimension of 3.6 mm [18]. Our work complements Ref. [17] by presenting results captured by a direct ToF sensor with improved timing resolution that features 128 histogram bins at a timing resolution of 200 ps per bin. We evaluate classification over a range of sample-to-sensor distances so that a classification system that is robust to distance is created. Further, since only a single ROI is used, the spatial dependence of the optical transient is not required for classification. Figure 1 displays photon return histograms captured by the AMS TMF8801 from a semi-translucent material (HPDE) and a material without subsurface scattering (stainless steel). The HDPE histogram shows a fast rising-edge from directly scattered light and a slower falling edge from light that penetrates and scatters within the material. In this work, we demonstrate that the relative intensity of direct reflection versus subsurface scattering coupled with the time delay of the subsurface response conveys sufficient information about the optical parameters of a material for classification. Our study classifies five plastic types using these raw ToF histograms captured over a range of distances. Successful classification using histogram data (0.96 accuracy) is followed by an investigation of classification success using three optical parameters extracted from a fit of the histogram data to a physics-based model. When only three extracted optical parameters are used as classifier features, classification accuracy degrades, but not dramatically so, and the likelihood that classification relies upon an unknown data artifact is minimized. The plastics measured are visually similar with comparable textures, and are sorted from multiple distances to validate the robustness of our approach.

## 2. Methods

### 2.1. Sensor System and Data Acquisition

**Figure 2:**
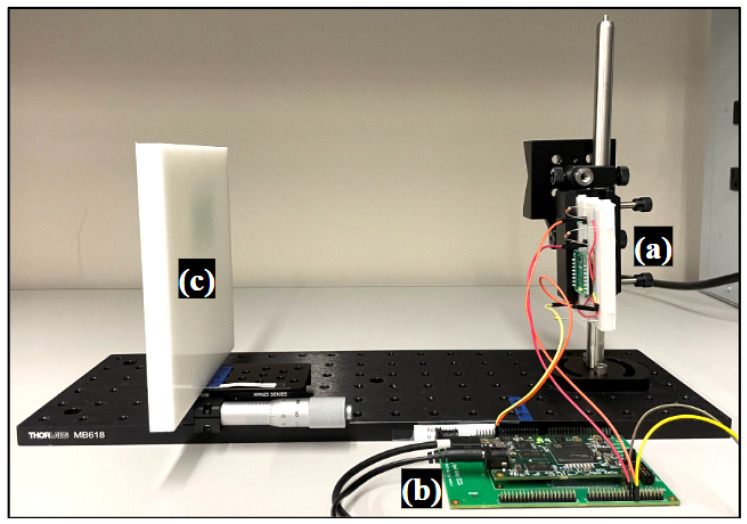
(**a**) Shows the evaluation board with the TMF8801, (**b**) the FPGA, and (**c**) the HDPE sample which demonstrates material placement with respect to the sensor.

The sensor system was composed of the AMS TMF8801 time-of-flight (ToF) sensor [18], mounted to the MIKROE LightRanger 5 Click evaluation board. An Opal Kelly XEM7310 FPGA (Xilinx Artix-7 FPGA) module with a USB-3 host interface was used to configure and read the ToF sensor (Figure 2). The FPGA design was derived from Ref. [19] and the host software leverages a Python package pyripherals, which organizes chip registers and abstracts the low-level Opal Kelly API calls [20]. The TMF8801 ToF sensor is a 4 × 32 single-photon avalanche diode (SPAD) array that captures a $\pm 9.5{}^{\circ }$ field of view (FoV) in long-range mode and $\pm 18.5{}^{\circ }$ in short-range mode with a recommended object range that extends from 2 cm to 250 cm. We configured the sensor to long-range mode to minimize the FoV so that the simple filled the field of the sensor and to minimize the histogram spread due to oblique photon paths. Data were downloaded from each of the five time-to-digital converters (TDCs) via burst I2C, which included a reference (TDC0) and four others (TDC1-4), each of which are connected to a different grouping of SPADs within the array, and therefore gather information from different spatial zones. Each raw histogram includes 128 time bins with each bin spanning 200 ps for a total time span of 25.6 ns, equivalent to an object depth of 3.84 m. Histogram readout instructions followed the sensor host driver communication application note including a requirement for I2C reads to occur in 128 byte blocks [21]. For rapid USB transfers, an Opal Kelly transfer mechanism of a USB PipeOut was combined with a FIFO buffer on the FPGA so that transfers proceeded in bursts to minimize the time needed for data transfer. Histogram samples were taken in a dark room to minimize ambient light.

### 2.2. Measurements

**Figure 3:**
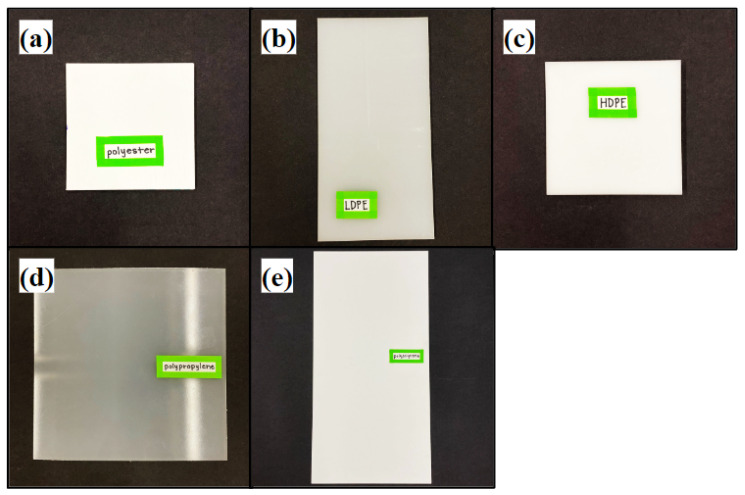
The materials under test are (**a**) polyester, (**b**) LDPE, (**c**) HDPE, (**d**) polypropylene, and (**e**) polystyrene.

For *sliding distance* classification studies, the object distance started at 4 cm from the sensor and was moved in small increments up to a distance of 24 cm from the sensor. The maximum distance of 24 cm was chosen based on the size of the materials and the FOV of the TMF8801 to ensure that the entire FOV was filled by the plastic sample under test, and did not include background materials. The closest edge of the measurement range was chosen to be outside the minimum distance the sensor can read accurately, which is 2 cm. For classification evaluation, the sensor was configured for 1800 k laser iterations, which corresponds to an estimated total ranging time of 46.1 ms. The sensor period was set to 128 ms between histograms with 100 histograms measured per sample. From the sensor datasheet, a period of 33 ms is possible with up to 900,000 iterations [18] such that 1800 k iterations allows for 15 histograms per second. *Fixed distance* measurements were captured with the samples 22.5 cm from the sensor to evaluate the relative significance of Poisson statistics in comparison to other sources of variation, including the object distance, surface normal, and object position within the field-of-view of the sensor. These experiments used various configurations of laser pulses per histogram with 200 histograms captured for each configuration and sample. The materials measured with McMaster–Carr part numbers in parentheses were polyester (8597K92), LDPE (8657K331), HDPE (8619K611), polypropylene (8742K133), and polystyrene (8734K34) (see Figure 3). Note that some of these samples have white colorants such that our results should not be generalized to samples of a specific plastic type.

### 2.3. Histogram Processing

Each raw histogram contains information from every TDC, and each is composed of two channels, channel 1 and channel 2. Both channels include 128 time bins of data. In preprocessing, we limit data to just channel 2 because the channel 2 histograms were of greater magnitude than channel 1, and since the TMF8801 datasheet is unclear on the distinction between the two channels. The histograms of TDCs 1–4 in channel 2 were summed, while the reference TDC0 was discarded. Because one objective of this work is to identify materials from any distance, the parameter of distance was eliminated. To do this, the index of the histogram peak was located and only five time bins on each side of the peak were retained. Classifier features were limited to 11 time bins to prevent overfitting with classifier models and to avoid reaching the maximum iterations when using a logistic regression model. When this limit is reached, parameter convergence becomes impossible, and the network training fails completely.

### 2.4. Optical Parameter Model

As an alternative to raw histogram data, the time-of-flight histogram data is parameterized by a physics-based model. The model includes a surface scattering term, a term from diffusive subsurface scattering, and background illumination. Surface scattering, in which the light does not penetrate the material, arises from Fresnel reflection with the outgoing angular distribution ranging from specular to Lambertian, and is determined by surface roughness. On the other hand, subsurface scattered light penetrates a translucent material, scatters in the bulk of the material (possibly multiple times), and reemerges sometime later at a different position and angle [22]. Light transport in the bulk of a material may be modeled completely using radiative transport equations, or simplified using assumptions, such as single scattering or diffusion [23]. In this work, we test optically thick samples and use a diffusion approximation to model subsurface scattering. We model surface scattering as a delta function spread in time by the Gaussian instrument response function (σ) (due to laser timing spread, the SPAD timing jitter, and the TDC timing jitter). The subsurface light transport and scattering is modeled by a Gaussian spread modified by an exponential decay (from optical diffusion and absorption within the material). The work of Heide et al. [24] models the material impulse response (MIRF) as a mixture of multiple exponentially modified Gaussians, each with a separate time center ($t_{0}$), standard deviation (σ), exponential decay (τ), and amplitude (*A*). Our model shown in Equation (1) follows and simplifies this approach by including a single surface term that is not modified by an exponential decay and a single subsurface term. The temporal spread (σ) is assumed to be dominated by the instrument response function such that this parameter is the same for both the direct reflection and subsurface scattering terms, similarly, a single center ($t_{0}$) as the samples are assumed to be strongly scattering. The two terms have independent amplitude parameters, $A_{1}$ quantifies direct surface reflection, and $A_{2}$ quantifies subsurface scattering.

(1) $$\begin{array}{c} \mathrm{MIRF}\left(t\right)=\frac{A_{1}}{\sqrt{2\pi }\sigma }\;\cdot \;\exp\left(-\frac{{\left(t-t_{0}\right)}^{2}}{2\sigma^{2}}\right)+\\ \;\frac{A_{2}}{2\tau }\exp\left(\frac{1}{2}{\left(\frac{\sigma }{\tau }\right)}^{2}-\left(t-t_{0}\right)/\tau \right)\;\cdot \;\left(1+\mathrm{erf}\left(\frac{t-t_{0}-\sigma^{2}\tau }{\sqrt{2}\sigma }\right)\right)+b \end{array}$$

**Figure 4:**
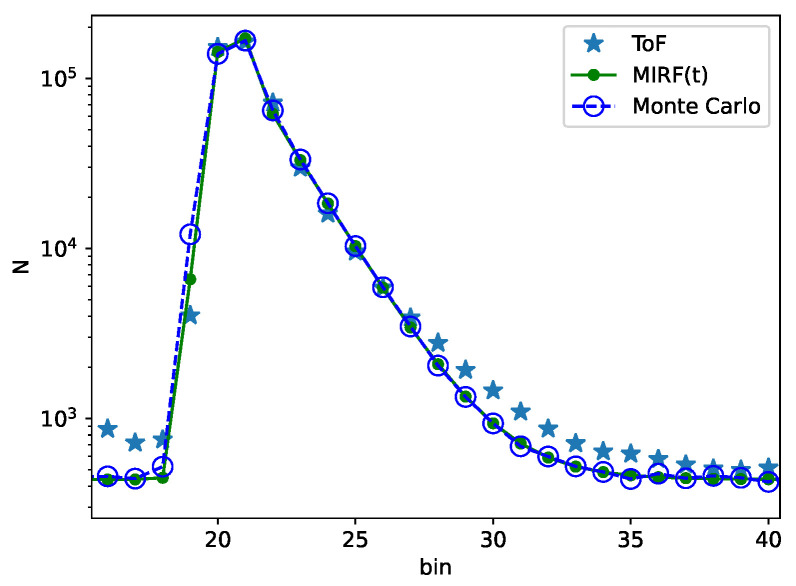
A measured response histogram, the result of a fit of the measured values to Equation (1), and a Monte Carlo simulation that regenerates the histogram curve using the extracted fit parameters.

Fits were limited to 10 bins to the left and 30 bins to the right of the bin with the maximum signal. The ToF sensor instrument response function (IRF) was determined separately and fixed to σ=0.55 bins during fits so that variations of the exponential response due to subsurface scattering would not be partially captured by changes in the IRF. Fits of 500 histograms (100 per sample) showed an average of $R^{2}=0.9999998$ and a minimum of $R^{2}=0.9999989$. Figure 4 demonstrates an example histogram from a plastic sample. The concordance of the data to the fit is clear, particularly in the regions of the exponential decay from the peak bin to bin 27. Beyond bin 27, the vertical log scaling of Figure 4 accentuates slight discrepancies, which suggests that a second subsurface scattering term and time constant (i.e., $\tau_{2}$) would further improve the fit.

### 2.5. Classifier Creation

TensorFlow, a Python-based machine learning platform, was used to create classifiers. Two separate classifiers were investigated with different input features. The first used 11 bins of processed histogram data, and the other used optical parameters extracted from the processed histogram data. The optical parameter fits were constrained to three features: the time constant of the subsurface scattering (τ), the ratio of the direct reflection and subsurface intensities ($A_{1}/A_{2}$), and the return time ($t_{0}$). Both types of feature data were randomly split into training and testing groups (80% was used to train, the other 20% for testing). Features were normalized using StandardScaler from sklearn.preprocessing to minimize the scale of the sample set and prevent imbalance when feature data were combined. Seven learning models were imported from *sklearn* and evaluated: logistic regression, k-nearest neighbor (kNN), support vector classification using both linear and radial basis function kernels (linear SVC and RBF SVC), Gaussian naïve Bayes (Gaussian NB), decision tree, and random forest. Each of these algorithms was trained and tested on the same dataset. The accuracy of the models on the test set was assessed using the multilabel accuracy score and confusion matrices that detailed success in sorting each material. The multilabel accuracy score is the fraction of true classifications as compared to total classifications.

## 3. Results

### 3.1. Sliding Distance Classification with Histogram Data

**Figure 5:**
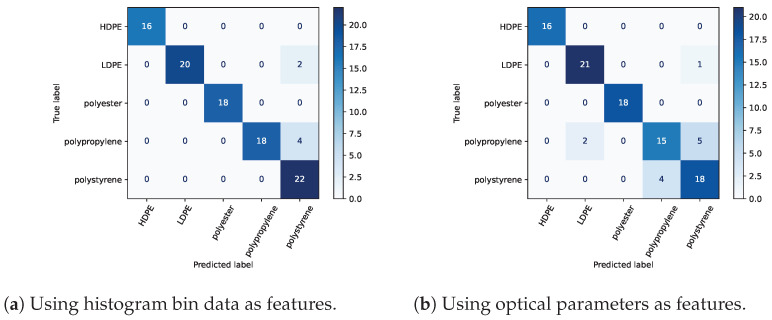
Confusion matrices of the RBF SVC using two different sets of features. Each material was measured 100 times and an average of 20 measurements per material were used for testing. For the two sets of features, the classifiers were retrained and then tested.

For both sets of features, the RBF SVC using a regularization parameter of 10 performed best in terms of the classification accuracy of the test set. The RBF SVC accuracy score was 0.96 using features of 11 histogram bins centered around the bin of maximum counts. The next most accurate models were linear SVC followed by logistic regression (accuracy scores of 0.92 and 0.87, respectively). The confusion matrix of Figure 5a shows the most common error to be incorrectly classifying polypropylene as polystyrene.

### 3.2. Sliding Distance Classification with Optical Parameters

**Figure 6:**
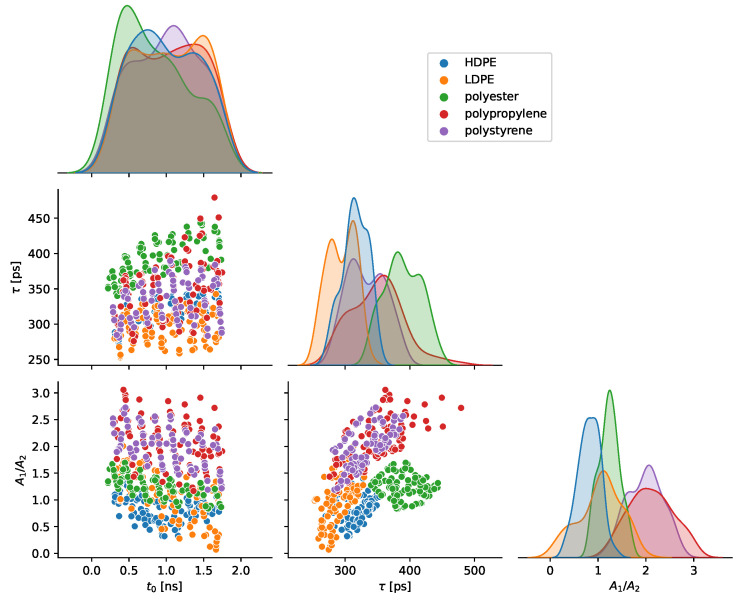
A pair plot of the three optical parameter features used for classification. Pairwise relationships are shown on the off-diagonals. The distributions of each individual parameter are shown on the diagonals. The histograms of $t_{0}$ show that all materials were measured through a nearly equivalent range of distances (∼4 cm to 24 cm). Minimal trends versus the object distance are seen for τ and $A_{1}/A_{2}$. By eye, materials sort well by τ and $A_{1}/A_{2}$, except for considerable similarities between polypropylene and polystyrene.

An RBF SVC classifier achieved an accuracy score of 0.88 using three optical parameter features of $t_{0}$, τ, and $A_{1}/A_{2}$. Figure 6 demonstrates the pairwise relationships for the three optical parameter features for the five materials under test. The distributions of individual parameters are shown on the diagonals. For example, the $t_{0}$ distributions on the top-left diagonal of Figure 6 show that the materials were smoothly varied through a range of distances. The scatter plot of $A_{1}/A_{2}$ versus τ shows groupings amenable to classification except for considerable overlap of polypropylene and polystyrene. This overlap manifests in the feature confusion matrix of Figure 5b as 9 of 12 total misclassifications are mislabels of polypropylene as polystyrene or vice versa. Figure 6 shows the dependence of τ and $A_{1}/A_{2}$ versus the return time $t_{0}$, which is proportional to object distance. These two parameters were selected partially due to anticipated independence on the object distance. Minimal trends are observed; the most apparent is τ increasing with $t_{0}$. A portion of this increase has a geometrical explanation. If the distance from the sensor to the point of the object in the center of the FoV is defined as *d*, then the distance from an object point at the edge of the FoV is d/cosθ, where θ is the half-angle of the FoV. The distance difference between these two object points is thus Δd=2d(1/cosθ−1), which for our system equates to 22 ps of additional ToF, given a 24 cm distance and a $\pm 9.5{}^{\circ }$ FoV.

The importance of each of the optical parameters was determined by permuting each feature using the sklearn function *permutation_importance*. Table 1 shows the results with the exponential decay time constant, τ, and the ratio of direct reflection to subsurface scattering, $A_{1}/A_{2}$, carrying nearly equal importance. The return time, $t_{0}$, was the least important feature. Models were trained and evaluated without $t_{0}$ as a feature, but classification accuracy was considerably degraded.

### 3.3. Fixed-Distance Classification with Optical Parameters

**Figure 7:**
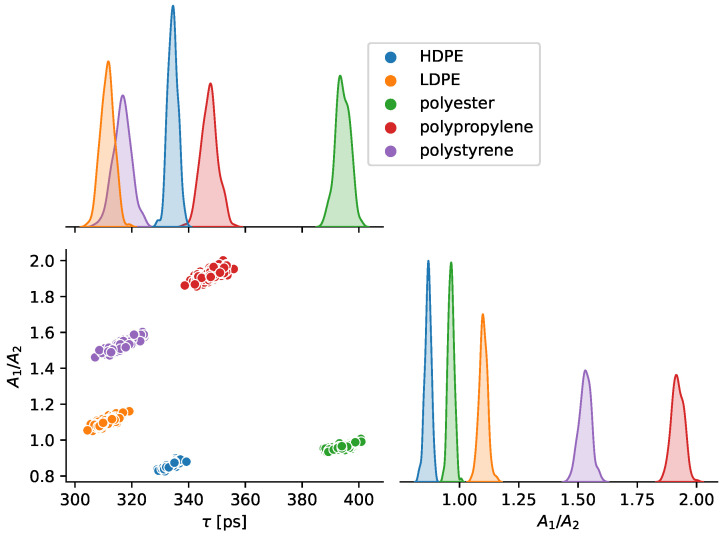
A pair plot of the the two optical parameter features used for plastic classification with repeated measurements at a fixed distance of 22.5 cm. Pairwise relationships are shown on the off-diagonals. The distributions of each individual parameter are shown on the diagonals. Each measurement was repeated 200 times with 600,000 laser pulses per histogram. The parameter $t_{0}$ was omitted, since it was constant and nearly equal for each sample.

The measurements at a fixed distance using only the features of τ and $A_{1}/A_{2}$ are summarized in Figure 7. For classifier training and data presentation, $t_{0}$ is removed, as the sample distance is fixed and the slight but consistent and measurable variations from sample to sample would clearly distinguish each plastic. Using these two optical parameters, a RBF support vector classifier was trained (800 measurements) and tested (200 measurements). As suggested by the clustering of Figure 7 and shown in the results of Table 2, there are no misclassifications at a fixed distance (other classifier models also showed perfect classification with this dataset). We assume that variation in the optical parameters captured in the fixed distance measurements was dominated by Poisson statistics. Then, the excess variation of parameters seen in sliding distance measurements must be due to sources of error other than Poisson statistics. The sliding distance results have larger standard deviations of τ and $A_{1}/A_{2}$ by factors of ×11.2 and ×16.6, respectively, as compared to the fixed distance measurements. This considerable discrepancy demonstrates that Poisson statistics is not the dominant source of variation in the sliding distance classification experiments. Rather, the variations in parameters are possibly due to differences in the distance, the object location within the FoV, and/or the angle of the object normal. The fixed-distance histograms were recreated by Monte Carlo simulations (Figure 4). The Monte Carlo simulations randomly sampled the number of photon counts in each histogram bin from a Poisson distribution with the average counts set by fits to the experimental data. Multiple histograms were generated using Monte Carlo simulations and then fit to the optical parameter model, so that the variation in each optical parameter caused by Poisson statistics could be determined. However, we did not explore the Monte Carlo simulations in detail, since photon noise was found to be an insignificant contributor to parameter variation in the sliding distance experiments.

## 4. Discussion

When the sample distance was varied, classification using raw histogram bin values as features outperformed three optical parameters with accuracy scores of 0.96 versus 0.87, respectively. The reduced classification accuracy indicates that the optical parameters do not fully capture the information in the transient returns for classification. Future work should investigate the minimal number of optical parameter features needed for classification success equivalent to raw histogram bins.

The optical parameter model assumes planar objects that are normal to the optical axis. A challenge, addressable by pixelated ToF sensors and minimized by small pixel FoVs, is the differentiation between subsurface scattering delays and the spreading of the transient return due to object tilt. Transient returns captured by the AMS TMF8801 were shown to allow inference of the object tilt [25]. Another model simplification instance is when materials are considered highly scattered and diffusive. Future work could include considerations for optically thin materials by including material thickness as an optical parameter and classifier feature. Other materials with different optical responses, such as highly translucent or predominately specular reflection, should be characterized with our methods. We captured transient data from a stainless steel (specular) plate that returned $A_{1}/A_{2}\approx 3.5$ which, as expected, is a larger ratio of direct reflection to subsurface scattering than the measured plastics ($A_{1}/A_{2}<2$ for all fixed distance measurements).

All of our results were captured using a single ToF device. An important question is whether the results would translate to another copy of the same ToF model so that a single classifier and training could be used for many sensors. The results of Figure 7 show that the sample τ varies meaningfully across materials by a value of nearly 1/2 a histogram bin, suggesting that data captured by another part would be similar. However, the falling edge of the transient return may be partially convolved with and obscured by the SPAD jitter timing tail [26], which likely varies from part to part.

Physics-based optical parameter features limit potential paths for data leakage. Leakage is when a spurious relationship between the captured data and the classification labels is present [27]. This relationship between the data and label will not persist when the system is redeployed in another environment such that experiments with data leakage overestimate real-world performance. A hypothetical and specific example for time-of-flight histograms would be if the background light slowly increases during data collection while sample measurements are collected in order. A classifier with raw histogram data features may sort based on this spurious relationship between sample and background counts, whereas classification using physics-based features may knowingly omit the ambient intensity. The approach of optical parameters as features was taken to reduce the chances of classification by unforeseen attributes or biases.

## 5. Conclusions

This work showed that an inexpensive direct ToF sensor can successfully classify optically similar plastics over a range of sensor-to-object distances. These methods allow for the possibility of material classification by a modestly cooperative smartphone user. This project was designed in response to the environmental crisis of plastic waste: the plastics measured are all recyclable, with resin codes 1, 2, 4, 5, and 6. Only 9% of plastic waste is recycled [28], and there exists extremely limited information on its natural breakdown, so it is critical that people shine a light on the consequences of its production. Because sensors such as the TMF8801 reside in the majority of smartphones, this classification technology could be publicly deployed. Plastic distinction increases the accuracy of recycling and may also compel people to see their everyday products through a new lens and inspire the purchase of eco-friendly alternatives. Beyond sustainability, non-contact material classification has a multitude of applications. Consider security uses, such as ensuring that a real finger is placed on a fingerprint sensor, or verifying that money is genuine. In the pursuit of self-driving cars or humanoid AI, ToF sensing could discern slightly different terrains and adjust motor operations accordingly. Textures and translucency in photography, special effects, and computer graphics rendering [23] may become more life-like and accurate through direct ToF-based optical parameter extraction. These highlight a few of the extensive ways that a miniaturized ToF-based material sensing system could be utilized.

## Figures and Tables

**Table 1:** Feature importance extracted by permuting features using *permutation_importance* from the sklearn module inspection.

$t_{0}$	τ	$A_{1}/A_{2}$
0.24	0.516	0.528

**Table 2:** A summary of the number of photons collected versus the TMF8801 datasheet parameter iterations, which is assumed to be the number of laser pulses. With samples at a fixed distance, the classification accuracy is perfect (using 80% train and 20% test). Per the datasheet, a total of 900,000 iterations or fewer supports a rate of at least 30 histograms per second [18].

Laser Pulses	Total Photons/Hist.	Classification Accuracy
100 k	58,816.8	1.0
300 k	162,887.3	1.0
600 k	319,091.8	1.0

## Data Availability

The data presented in this study are available on request from the corresponding author.

## Abbreviations

The following abbreviations are used in this manuscript:

ToF	Time of flight
RGB	red, green, blue
MIRF	material impulse response function
TPSF	temporal point spread function
TDC	time-to-digital converter
PMD	photon mixing device
ROI	region of interest
SPAD	single photon avalanche diode
LDPE	low-density polyethylene
HDPE	high-density polyethylene
A.I.	artificial intelligence
k-NN	k-nearest neighbor
NB	naive Bayes
SVC	support vector classifier
RBF	radial basis function
