# Correction: Becker, C.N.; Koerner, L.J. Plastic Classification Using Optical Parameter Features Measured with the TMF8801 Direct Time-of-Flight Depth Sensor. *Sensors* 2023, *23*, 3324

**DOI:** 10.3390/s23187986

**Published:** 2023-09-20

**Authors:** Cienna N. Becker, Lucas J. Koerner

**Affiliations:** Department of Computer and Electrical Engineering, University of St. Thomas School of Engineering, St. Paul, MN 55105, USA; cienna.becker@stthomas.edu

## Text Correction

There was an error in the original publication [1]. Equation (1) was incorrectly typeset. The last term in the numerator of the error function (erf) should be *σ*^2^/*τ*.

A correction has been made to Section 2. Methods, Section 2.4. Optical Parameter Model, Equation (1). The correct equation is:

(1) $$\begin{array}{c} MIRF\left(t\right)=\frac{A_{1}}{\sqrt{2\pi }\sigma }\cdot \exp\left(-\frac{{\left(t-t_{0}\right)}^{2}}{2\sigma^{2}}\right)+\\ \;\frac{A_{2}}{2\tau }\exp\left(\frac{1}{2}{\left(\frac{\sigma }{\tau }\right)}^{2}-\left(t-t_{0}\right)/\tau \right)\cdot \left(1+\mathrm{erf}\left(\frac{t-t_{0}-\sigma^{2}/\tau }{\sqrt{2}\sigma }\right)\right)+b \end{array}$$

The authors state that the scientific conclusions are unaffected. This correction was approved by the Academic Editor. The original publication has also been updated.
